# Segmental Helical Motions and Dynamical Asymmetry Modulate Histidine Kinase Autophosphorylation

**DOI:** 10.1371/journal.pbio.1001776

**Published:** 2014-01-28

**Authors:** Ariel E. Mechaly, Nathalie Sassoon, Jean-Michel Betton, Pedro M. Alzari

**Affiliations:** Institut Pasteur, Unité de Microbiologie Structurale and CNRS UMR 3528, Paris, France; Princeton University, United States of America

## Abstract

Histidine kinases (HKs) are dimeric receptors that participate in most adaptive responses to environmental changes in prokaryotes. Although it is well established that stimulus perception triggers autophosphorylation in many HKs, little is known on how the input signal propagates through the HAMP domain to control the transient interaction between the histidine-containing and ATP-binding domains during the catalytic reaction. Here we report crystal structures of the full cytoplasmic region of CpxA, a prototypical HK involved in *Escherichia coli* response to envelope stress. The structural ensemble, which includes the Michaelis complex, unveils HK activation as a highly dynamic process, in which HAMP modulates the segmental mobility of the central HK α-helices to promote a strong conformational and dynamical asymmetry that characterizes the kinase-active state. A mechanical model based on our structural and biochemical data provides insights into HAMP-mediated signal transduction, the autophosphorylation reaction mechanism, and the symmetry-dependent control of HK kinase/phosphatase functional states.

## Abstract 2

Step-by-step structures of a prototypical *E. coli* histidine kinase reveal how external stimuli drive helical bending motions to control asymmetric movements of the catalytic domains.

## Author Summary

Bacteria use two-component signal transduction systems (TCSs), composed of a histidine kinase (HK) and a downstream response regulator (RR), to perceive a broad range of external stimuli and trigger an appropriate response. Prototypical HKs are transmembrane homodimeric receptors with an external sensor region coupled to a cytoplasmic catalytic core through an adaptor HAMP domain. By a mechanism that is still poorly understood, the external input signal propagates through the HAMP domain to control the activity of the catalytic domain, which adds a phosphate tag, first to the HK itself (“autophosphorylation”), ready for subsequent transfer to the RR. In this study, we report the first detailed structures of the full cytoplasmic domain of a prototypical HK, CpxA, which is involved in the detection and response to damage of the cell envelope of Escherichia coli. The structures provide snapshots of different kinase states along the autophosphorylation reaction. Our combined genetic, biochemical, and structural data point to a mechanical model for HK activation in which the input signal induces coordinated helical motions that regulate catalysis by asymmetrically repositioning the catalytic domains.

## Introduction

Bacteria commonly use two-component signal transduction pathways to couple environmental stimuli to adaptive responses [1]. Typically composed of a sensor histidine kinase (HK) and a response regulator (RR), two-component systems (TCSs) are abundant in bacterial genomes, reflecting the diversity of stimuli that bacteria are capable of perceiving and responding to [2]–[4]. Independently of the nature of input signals, TCSs share a common signal transduction mechanism [3]. Stimulus perception triggers a two-step phosphorylation cascade consisting in the autophosphorylation of the HK at a conserved histidine residue followed by the transference of the phosphoryl group to an aspartate in the cognate RR, usually a transcription factor that initiates the bacterial response by modifying target gene(s) expression.

HKs are homodimeric receptors with a modular architecture, which in its simplest form consists of a sensor domain and a cytoplasmic transmitter core. Whereas the signal-dependent sensor domains are highly variable, the transmitter core is more conserved. It contains a dimerization and histidine phosphotransfer (DHp) domain coupled to a catalytic ATP-binding (CA) domain. Mainly depending on the cellular location of the sensor domain (either extracytoplasmic, membrane-anchored, or cytosolic), a wide variety of HK architectures exists [4],[5]. However, the prevalent form of HKs is a transmembrane receptor with an extracytoplasmic sensor domain coupled to the cytoplasmic catalytic core through an adaptor HAMP domain [6], a small protein module that is commonly found in Histidine kinases, Adenyl cyclases, Methyl-accepting chemotaxis proteins and Phosphatases. During the last few years, a number of structural studies have shed light into various aspects of the TCS mechanism of signal transduction. Crystal structures of isolated HK sensor domains have revealed the conformational consequences of stimulus perception [7]–[9], whereas those of HK transmitter cores illustrated the conversion between different functional states through DHp interhelical rearrangements [10],[11], and two structures of cytoplasmic HK–RR complexes provided the structural basis underlying the RR dephosphosphorylation reaction [12],[13]. Nevertheless, some important questions on TCS signal transduction and autophosphorylation mechanisms remain largely unanswered. For instance, it is poorly understood how a stimuli-induced symmetry/asymmetry switch regulates the HK catalytic activities. Moreover, a full structural characterization of the autophosphorylation reaction remains elusive, possibly hampered by the intrinsic flexibility and highly dynamic nature of the catalytic core [10], as well as by the transient nature of the reaction intermediate in HKs [14]. There is also a lack of structural data on prototypic (HAMP-containing) HKs. So far, none of the HKs for which the transmitter core has been crystallized [10]–[13],[15] belong to the predominant HK subfamily containing HAMP domains directly linked to the catalytic core [4].

To gain insight into the mode of action of prototypical HKs, we chose to focus on *Escherichia coli* CpxA, the HK component of the Cpx signaling system that regulates an envelope stress response in *E. coli* [16],[17]. The sensor kinase CpxA senses a variety of envelope stresses, including misfolded proteins, and transduces this signal to the RR CpxR through conserved phosphotransfer reactions. Phosphorylated CpxR then activates transcription of genes whose products are involved in protein physiology in the envelope [18]. Cpx regulon includes a large number of members [19], mainly periplasmic chaperones and proteases [20]–[23]. In addition to CpxA and CpxR, the Cpx system includes the auxiliary periplamic protein CpxP, a dimeric α-helical protein [24],[25] that could interact with the CpxA periplasmic sensor domain and was shown to tightly regulate autokinase activity *in vivo* [26] and *in vitro* [27]. Here we report the 3D structures of the full cytoplasmic region of CpxA in several different crystal forms. These structures show CpxA adopting different asymmetric conformations, including a state that corresponds to the autophosphorylating Michaelis complex and provides insights into the reaction mechanism. The ensemble of structures illustrates the highly dynamic and strongly asymmetric nature of the HK kinase-active state. Based on our mutagenesis, structural, and biochemical data, we propose a mechanical model in which propagation of the conformational signal through the HAMP domain can regulate autokinase activity by inducing segmental movements and pronounced bending of the central HK α-helices, which in turn control the mobility of the ATP-binding catalytic domains.

## Results and Discussion

### Overall Structure of the CpxA Transmitter Core

**Figure 1:**
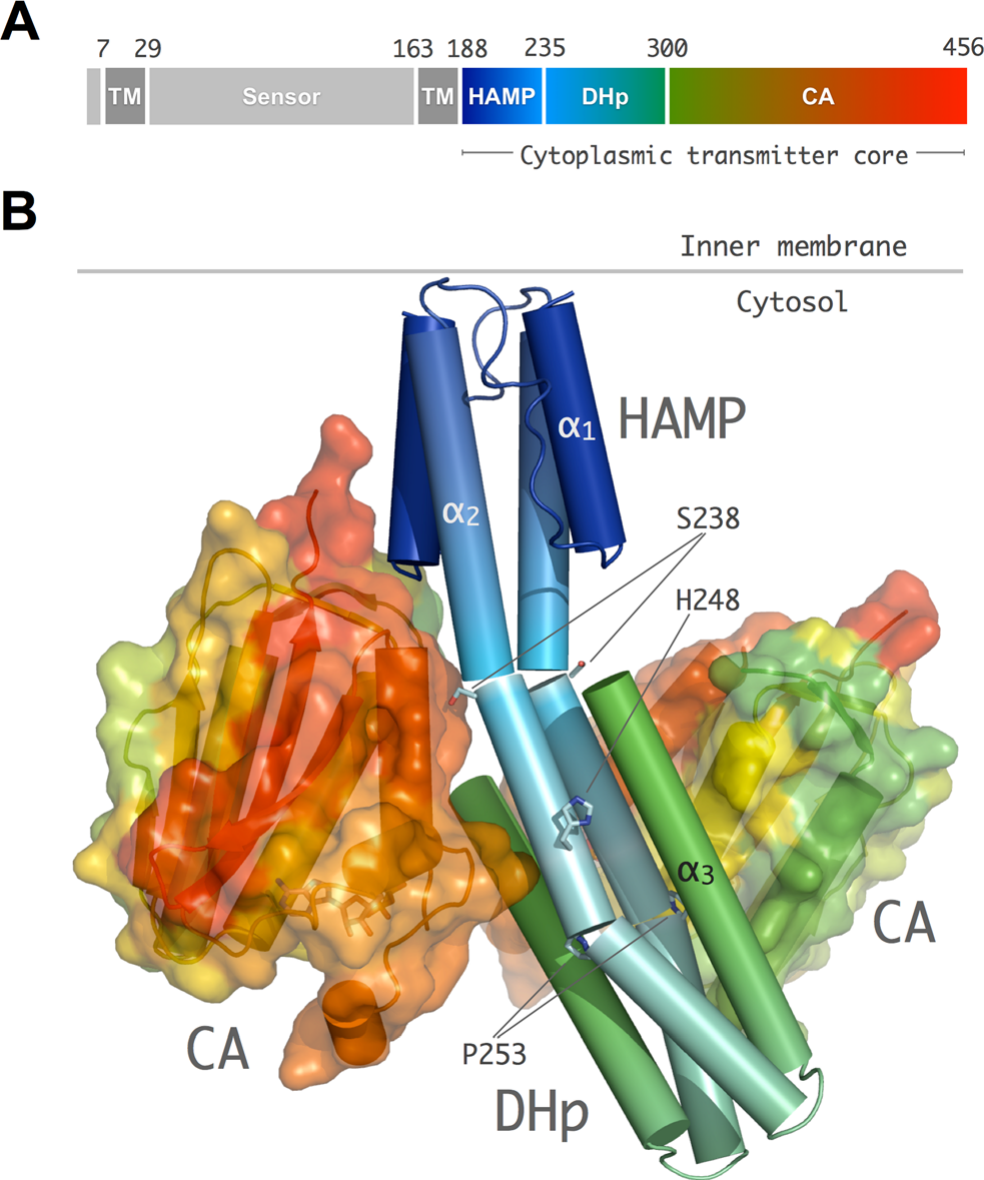
Overall structure of CpxA. (A) Linear representation of the prototypical CpxA domain organization. CpxA is an integral membrane receptor with a periplasmic sensor region (residues 29 to 163) flanked by two transmembrane helices (TM1 and TM2). TM2 connects the sensor domain to the cytosolic transmitter core (residues 188 to 457) formed by three domains: HAMP, DHp, and CA, rainbow colored from N-terminus–C-terminus (blue-red). (B) Cartoon representation of the CpxA_HDC_ homodimer in the trigonal crystal form. The homodimer is highly asymmetric due to helical kinks nearby Ser238 and Pro253 in helix α_2_ (shown in stick representation) and large differences in the positioning and orientation of the two CA domains (shown in surface representation) with respect to the central DHp helical core.

The full cytoplasmic region of *E. coli* CpxA (CpxA_HDC_), encompassing the HAMP signaling domain (residues 188–237) and the DHp and CA domains of the catalytic core (residues 238–457), has been produced as recombinant protein for structural studies (Figure 1A). The 3D structures of wild-type CpxA_HDC_ (or the point mutant CpxA_HDC_M228V_) in complex with nucleotides have been determined in five distinct crystalline environments (Table 1) using either single-wavelength anomalous diffraction (SAD) or molecular replacement methods (see Materials and Methods). The CpxA_HDC_ structure consists of a homodimer, in which the HAMP and DHp domains from both subunits assemble into an elongated central coiled-coil region flanked on either side by the two CA domains (Figure 1B).

**Table 1:** Crystallographic data collection and refinement statistics.

Data Collection and Refinement	CpxA_HDC_-ADP	CpxA_HDC_-ADP	CpxA_HDC_-ATP	CpxA_HDC_M228V_-AMPPNP	CpxA_HDC_-ATP	CpxA_HDC_M228V_-ADP	CpxA_HDC_M228V_-ADP
*Data collection*							
Synchrotron beamline	ESRF ID29	ESRF ID29	SOLEIL Proxima 1	SOLEIL Proxima 1	SOLEIL Proxima 1	SOLEIL Proxima 1	SOLEIL Proxima 2
Dataset	Native	Pt-derivative	SeMet	Native	SeMet	Native	Native
Wavelength (Å)	1.072	1.072	0.979	0.980	0.979	0.991	0.980
Resolution range (Å)	47.92–3.65 (3.85–3.65)	49.03–4.10 (4.49–4.10)	46.42–3.40 (3.67–3.40)	47.29–2.85 (2.99–2.85)	49.21–3.30 (3.56–3.30)	47.46–2.00 (2.05–2.00)	48.43–3.30 (3.56–3.30)
Space group	C222_1_	C222_1_	P3_1_21	P6_1_22	P6_1_22	C2	I2
Unit cell							
a, b, c (Å)	143.2, 191.7, 205.0	139.1, 186.3, 195.1	141.8, 141.8, 121.6	144.3, 144.3, 250.5	145.2, 145.2, 246.1	95.1, 141.8, 55.2	91.75, 139.1, 114.1
α, β, γ (°)	90, 90, 90	90, 90, 90	90, 90, 120	90, 90, 120	90, 90, 120	90, 94.3, 90	90, 99.19, 90
R-sym	0.099 (1.733)	0.067 (0.695)	0.084 (2.277)	0.073 (1.020)	0.079 (0.808)	0.036 (0.452)	0.135 (0.742)
I/σ (I)	18.7 (1.9)	10.4 (1.9)	19.2 (2.1)	18.4 (2.1)	31.3 (5.3)	15.9 (2.4)	7.8 (2.4)
CC (1/2)	1.000 (0.685)	0.998 (0.770)	1.000 (0.886)	0.999 (0.691)	1.000 (0.980)	0.999 (0.878)	0.995 (0.777)
Completeness (%)	100.0 (100.0)	94.8 (96.0)	99.9 (99.9)	97.1 (98.3)	100.0 (100.0)	99.8 (100.0)	98.9 (95.8)
Multiplicity	13.5 (13.9)	3.8 (3.9)	13.0 (13.0)	11.0 (11.5)	25.1 (26.4)	3.8 (3.7)	3.7 (3.7)
*Refinement*							
Resolution (Å)	3.65		3.40	2.85	3.30	2.00	3.30
R-factor/R-free	0.210/0.227		0.227/0.233	0.207/0.213	0.204/0.229	0.194/0.216	0.200/0.218
Number of reflections	31,708		19,794	35,347	23,821	49,073	21,080
Number of atoms							
Protein	11,897		4,230	3,824	3,631	3,577	7,060
Ligands/ions	96		62	74	85	33	74
Water	0		0	19	0	305	0
B-factors (Å^2^)	188.25		185.49	104.38	189.89	57.58	109.10
RMS deviations							
Bond length (Å)	0.008		0.010	0.010	0.013	0.010	0.010
Bond angles (°)	0.96		1.20	1.16	1.89	1.05	1.10
PDB code	4BIU		4BIV	4BIW	4CB0	4BIX	4BIY

Values for the highest resolution shell are shown in parentheses.

The polypeptide chains of the HAMP domain were fully traced into SAD-phased experimental electron density maps of the CpxA_HDC_ orthorhombic and trigonal crystal forms (Figure S1). The domains are formed by two parallel helices (α_1_ and N-terminal part of α_2_) linked by a long connector (Figure 1B), which despite a low sequence identity adopt a dimeric four-helix bundle similar to the Af1503 and VicK HAMP structures (Figure S2) [15],[28]–[30]. In some crystal forms, however, only the C-terminal α_2_ helix from both monomers could be traced in the electron density maps, because CpxA_HDC_ has a tendency to establish interactions via its central-core helices, leading to the formation of dimers-of-dimers that sterically interfere with the positioning of helix α_1_ (Figure S3A). Indeed, previous biophysical studies of full-length CpxA reconstituted in nanodiscs had indicated that the protein could exist as a mixture of dimers and tetramers (Subrini, PhD thesis, 2011). We carried out SAXS studies of CpxA_HDC_ at different protein concentrations, which also demonstrate that CpxA_HDC_ is prone to associate into dimers-of-dimers, as scattering data suggest a concentration-dependent equilibrium between dimers and tetramers in solution (Figure S3). Guinier analysis shows no significant nonspecific protein aggregation, albeit the Rg dependence with protein concentration points to interparticle interactions that would likely favor the tetrameric state at protein concentrations similar to those used for the crystallization trials (>10 mg/ml). However, extrapolation of these data to very low concentration suggests that CpxA homodimers are the functionally relevant form of the protein, given the low protein abundance at the *E. coli* inner membrane.

The DHp antiparallel two-helix hairpins (helices α_2_ and α_3_) from both protomers also assemble into a four-helix bundle (Figure 1B) as observed in other HKs structures [10],[11],[31]. Both the HAMP and DHp coiled coils display the characteristic heptad repeats periodicity, in which the inner heptad positions *a* and *d* are occupied by hydrophobic residues. Besides the presence of a heptad repeat stutter at Leu232 [32], HAMP and DHp domains merge into a long continuous helix (α_2_) (Figure 1B), in a very similar way as previously reported for the chimeric fusion between AF1053 HAMP and DHp EnvZ [28]. At the C-terminus of each monomer, DHp helix α_3_ connects through a short flexible linker (residues 300–304) to the CA domain (Figure 1B). These catalytic domains adopt the ATP-binding Bergerat fold [33], a two-layer 3α/5β sandwich fold typical of the GHKL (bacterial gyrase, HSP90, histidine kinase, MutL) superfamily. In HKs, this topology is characterized by four conserved sequence motifs termed G1, G2, F, and N boxes (Figure S4) that include residues engaged in direct contacts with the bound ATP or confer flexibility to a flexible lid loop that surround the nucleotide binding cleft.

### Conformational and Dynamical Asymmetry of the CpxA Catalytic Core

The 3D structure of CpxA_HDC_ is highly asymmetric, not only by a different orientation of the CA domains, but also as a consequence of two potential bending points in the central helix α_2_ (Figure 1B). The first kink at Ser238 is located at the boundary between the HAMP and DHp domains, where there is a discontinuity between the parallel (HAMP) and the antiparallel (DHp) four-helix bundles. Helical bending at this position is probably required to avoid steric clashes at a layer of polar residues (Gln239–Gln240). The second kink is caused by the presence of a highly conserved proline residue (Pro253) in the proximity of the phosphorylatable His248, and has been also observed in other HK structures, such as HK853 [11], KinB [34], and VicK [15]. As a consequence of different bending angles in the two central α_3_ helices, the overall structure of the homodimer displays a curved asymmetric conformation (Figure S5), similar to that reported for the wild-type Af1503 HAMP–EnvZ DHp chimera [28]. It is interesting to note that the presence of two-helix–disrupting centers within α_2_ implies segmental helical movements in response to any torsional or orientational stress signal transmitted through the HAMP domain, which could easily result in asymmetric helical bending of the DHp domain.

**Figure 2:**
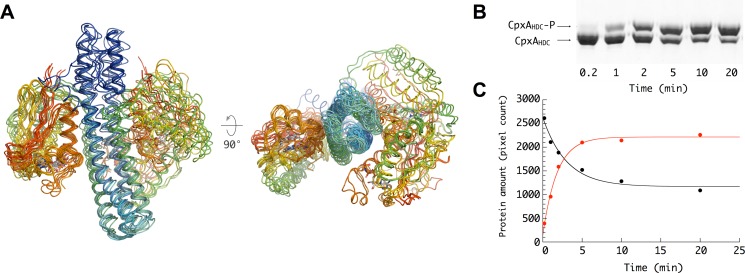
Conformational and dynamical asymmetry of the homodimer. (A) Superposition of all crystallographically independent CpxA_HDC_ dimers present in the five different crystal forms reported in this study. The main conformational difference between the distinct CpxA_HDC_ dimers consists in the position of one CA domain (left in the figure) relative to an invariant region containing both DHp domains and the second CA domain. (B) Phos-tag gel retardation autophosphorylation assay. The assay was performed with 10 µM CpxA_HDC_ and 1 mM ATP in 20 mM Hepes buffer (pH 7.6), 100 mM NaCl, 50 mM KCl, and 5 mM MgCl_2_ at 25°C. At the indicated time points, 15 µl aliquots were removed and mixed with SDS loading buffer. Phospho-proteins were separated by Phos-tag acrylamide gel electrophoresis. (C) Total amount of CpxA_HDC_-P and CpxA_HDC_ in each band was determined by densitometry analysis. The continuous lines were the best fits of the data to a single exponential term.

When considering together all CpxA_HDC_ crystal structures, it transpires that the DHp helical bending promotes a strikingly different dynamic behavior of the two CA domains in the homodimer, primarily due to distinct interactions with the central four-helix bundle. Thus, whereas one CA domain (from subunit A) is retained in a fixed—inactive—conformation close to the DHp helices (α_2_ from subunit B and α_3_ from subunit A) through a relatively large contact interface (∼1,200 Å^2^), the other CA domain with apparent unrestrained mobility adopts disparate orientations (Figure 2A) and displays significantly smaller CA–DHp surface contact areas, the largest of which (∼800 Å^2^) corresponds to the autophosphorylating Michaelis complex (see below). This strongly suggests a highly dynamic autophosphorylation process, leading primarily to a single phosphorylation event per homodimer, a feature already described for various HKs [10],[35],[36]. In fact, *in vitro* autokinase activity assays show that CpxA_HDC_ phosphorylation is never complete (Figure 2B), with only ∼70% of the protein phosphorylated at steady state (Figure 2C). These results suggest that CpxA could be mainly hemiphosphorylated *in vivo* under stress conditions, as the higher *in vitro* autophosphorylation efficiency might be attributed to subunit exchange during the assay, as observed for EnvZ [37].

### The Autophosphorylating Michaelis Complex

**Figure 3:**
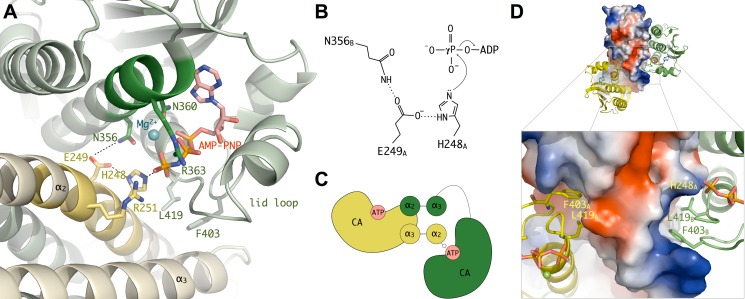
Autophosphorylating Michaelis complex. (A) Close view of the active site showing the residues directly involved in catalysis, as revealed by the hexagonal crystal structure of the CpxA_HDC_M228V_–AMPPNP complex (a very similar active site architecture was observed for the trigonal and hexagonal crystal structures of wild-type CpxA_HDC_ in complex with ATP, obtained at lower resolution). The regions corresponding to the conserved N and H boxes are highlighted in dark green and yellow, respectively. (B) Activation of His248 for phosphoryl transfer. Hydrogen bonding interactions between the imidazole ring of His248, the adjacent acidic residue in the CA domain (Glu249) acting as a general base, and a polar residue from the DHp domain (Asn356) contribute to activate His248 for nucleophilic attack to γ-P of ATP. (C) Schematic representation of the CpxA homodimer illustrating the *trans*-autophosphorylation reaction. (D) Anchoring of Phe403 and Leu419 to the DHp four-helix bundle represented by its electrostatic surface. The DHp-sequestered (yellow) and mobile (green) CA domains are shown in cartoon representation.

In all homodimers, the fixed CA domain is sequestered by the DHp domain in an inactive kinase conformation, with the ATP-γP positioned far away from the phosphorylatable His248. Instead, the active site formed by the second, mobile CA domain is poised for catalysis in three independent structures crystallized in two distinct crystal forms (trigonal and hexagonal space groups, Table 1), with the ATP γ-P and the acceptor His248 Nε atom well positioned for the phosphotransfer reaction to proceed (Figure 3A). The catalytic cores (DHp + CA domains) are very similar to each other in the three independent crystal structures (Figure S6); they can be superimposed with r.m.s.d. of 0.9–1.4 Å and display an identical network of hydrogen bonding interactions involving key functional residues for ATP binding and catalysis. This highly conserved active site geometry in distinct crystalline environments emphasizes the stability of the CA–DHp interface in the Michaelis complex and lends further support to its functional relevance.

Most residues interacting with the nucleotide belong to the CA domain (Figure S7), except for the phosphoacceptor His248 and the conserved Arg251, both of which make strong hydrogen bonding interactions with oxygen atoms from the ATP γ-P. The ATP phosphate groups are further stabilized in the binding pocket at the correct position for catalysis through additional contacts made by their oxygen atoms with the backbone amide nitrogen atoms of Leu419, Gly420, and Leu421, the hydroxyl groups of Thr417 and Tyr364, the guanidinium group of Arg363, and the carboxamide group of Asn360 (Figure 3A and Figure S7). Most of these residues belong to conserved sequence motifs in the HK family [38]; in particular, Asn360 is a strictly conserved residue from the N-box in the CA domain that is essential for the autophosphorylation reaction [39],[40].

The geometry of the active site points to Glu249, the amino acid adjacent to the phosphoacceptor His248 in the DHp domain, as a key functional residue. Its carboxylic side chain makes hydrogen-bonding interactions with both the Nδ atom of His248 and the carboxamide group of Asn356 from the CA domain (Figure 3A). These interactions favor the deprotonation of the His248 Nε atom, which performs the nucleophilic attack on the ATP γ-P (Figure 3B). Consistent with such a role, mutations of this glutamate were found to abolish kinase activity in other HKs such as NtrB [41] and CdrS [42], and an acidic residue at this position (immediately following the phosphoacceptor histidine) is highly conserved in major HK subfamilies HisKA (Pfam00512) and HisKA_3 (Pfam07730), which represents about 90% of all known HK sequences. Further evidence supporting a key functional role of this residue is provided by the structural comparison of the CpxA active site with that of the chemotaxis HK CheA [43]. Although the two proteins have a different topology, a CheA glutamic acid (Glu67) is spatially equivalent to CpxA Glu249 and interacts in an analogous way with the phosphoacceptor His45 (Figure S8), strongly suggesting a similar functional role for the acidic residue in the autophosphorylation mechanism.

Also important is the interaction between Glu249 and Asn356 across the DHp–CA interface (Figure 3A), which could help to achieve the correct disposition and polarization of Glu249 for catalysis. In agreement with this hypothesis, we found that the substitution Asn356–Tyr presented a strong kinase-deficient phenotype (Table 2) in a genetic screen designed to search for mutations that affect kinase activity *in vivo* (see below). It is interesting to note that a conserved glutamate at the equivalent position of CpxA Asn356 acts as a general base in the catalytic reaction of ATPases from the GHKL superfamily, which share a similar ATP-binding domain with HKs [44],[45].

**Table 2:** Identification of intragenic suppressors.

cpxA Allele	Residue Change	Location	β-Galactosidase Activity (Miller Units)
cpxAwt	—	—	582±25
cpxAΔP	Δ29–163	SD	13 523±250
cpxAC5	S185R	HAMP	22±5
cpxAA3	L186Q	HAMP	394±20
cpxAC51	N204Y	HAMP	102±15
cpxAA6	G222D	HAMP	69±10
cpxAA4	G222R	HAMP	135±25
cpxAB4	M228V	HAMP	5±2
cpxAC6	N356Y	CA	38±5

A plasmid encoding CpxA_ΔP_, a constitutive kinase CpxA variant lacking the sensor domain, was mutagenized and transformed into a *cpxA* null P_cpxP_–*lacZ recA* strain background. The table shows the CpxA activity conferred by single mutant *cpxA* alleles in the NS54 strain. Point mutations were identified from colonies displaying a Lac^−^ phenotype in X-gal containing plates.

In the Michaelis complex, the CA carrying the ATP molecule and the phosphorylatable His belong to different subunits of the homodimer, which structurally confirms *trans*-autophosphorylation for CpxA (Figure 3C). However, it is worth noting that *trans*-phosphorylation does not seem to be a generic feature of HKs; recent work by Ashenberg and coworkers [46] has shown that the *cis/trans* character of the reaction depends not on the local structure of the active site, but on the handedness of the DHp domain, which is determined by the loop at the base of the DHp four-helix bundle (i.e., between helices α2 and α3 in CpxA; Figure 1). This model is now further confirmed by the structural comparison of CpxA (*trans*-autotophosphorylation) and VicK (*cis*-autophosphorylation, [15]). Although both proteins have opposite handedness of the central four-helix bundle, their structures revealed a very similar active CA–DHp interaction (Figure S9).

In addition to the polar interactions described above, two hydrophobic residues from the CA domain (Phe403 in the F box and Leu419 in the G2 box) play a key dual role in stabilizing the DHp–CA interface for both the active and inactive CA conformations (Figure 3D). In the active conformation, the side chain of Leu419 is in contact with the phosphorylatable His248, while Phe403 interacts with Leu292 and the aliphatic moiety of Arg296 in DHp helix α3 from the opposite monomer. In the second, inactive, subunit the same two residues participate in anchoring the CA domain to a hydrophobic binding pocket mainly defined by residues Met287, Leu291, and Met 294 from helix α_3_ (in the same monomer) and residues Leu242, Ile246, and Leu250 from helix α_2_ (in the opposite monomer), accounting for a larger DHp–CA domain interface. Interestingly, some of these residues correspond to the *a* and *d* core positions of the DHp coiled-coil, which become partially accessible in one of the monomers due to the asymmetric conformation (see below).

These differences in the hydrophobic anchoring of Phe403 and Leu419 to the DHp domain (Figure 3D) might also contribute to modulate ATP binding affinity during the catalytic cycle. We have observed that the CpxA_HDC_ homodimer exhibited half-occupation of the active site in some crystal forms, and in these cases, only the less mobile CA domains (i.e., those sequestered by stronger hydrophobic interactions with the DHp domain) consistently displayed a bound nucleotide covered by the lid loop (Figure S10), whereas the CA domains with higher mobility have an empty nucleotide-binding cleft. These observations suggest that proper positioning of the lid loop through specific interactions with the DHp domain might trigger ATP binding. This hypothesis is consistent with recent observations from *Mycobacterium tuberculosis* DosS [47], which is unable to bind ATP until the CA domain is properly positioned for catalysis, and is reminiscent of previous findings on CheA dimers [48], where the two sites have largely different affinities for ATP. In our case, a lower nucleotide substrate affinity for the floppy CA domains might facilitate nucleotide turnover during the HK activation cycle.

### The HAMP Domain and CpxA Signal Transduction

**Figure 4:**
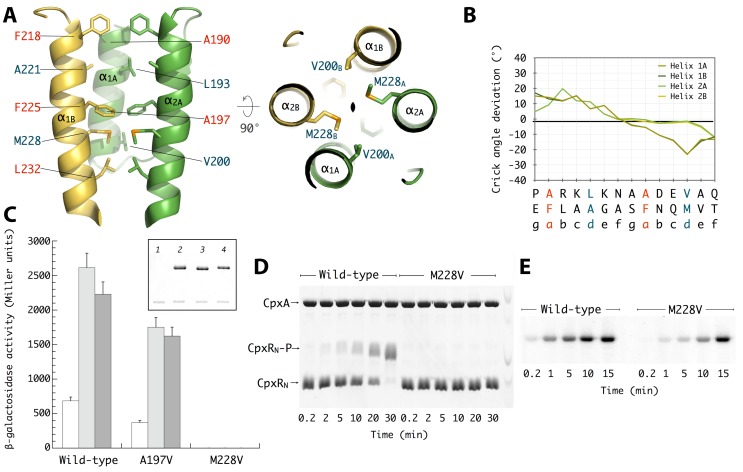
HAMP structure and function. (A) Side and top views of the dimeric HAMP four-helix bundle as observed in the trigonal crystal form. Core hydrophobic residues are shown in stick representation. (B) Crick angle deviation plot of the HAMP helices, as calculated with program samCC [52]. (C) The ability of wild-type and mutant *cpxA* bacteria to respond to the periplasmic overproduction of either the wild-type MalE (open bars) or the folding-defective mutant MalE31 (light gray bars) and to the presence of 0.2% phenethyl alcohol (dark gray bars) was monitored by measuring β-galactosidase activity from a *cpxP*–*lacZ* fusion contained in the NS54 strain expressing the different *cpxA* alleles. The insert shows the cellular levels of CpxA analyzed by immunobloting membrane protein fractions prepared from the NS54 strain transformed by pLCB (lane 1), pLCBA_wt_ (lane 2), pLCBA_197_ (lane 3), and pLCBA_228_ (lane 4). The additional band observed in the immunoblot is a cross-reacting protein recognized by the antiserum that serves as a loading control. (D) Phosphotransferase activity of CpxA. Both full-length CpxA and CpxA_M228V_ proteins (10 µM) were first allowed to autophosphorylate for 20 min at 25°C in the presence of 1 mM ATP, and then an equimolar amount of CpxR_N_ (N-terminal receiver domain) was added to the reactions. Samples were removed at the indicated time points, and phospho-proteins were separated by Phos-tag acrylamide gel electrophoresis. (E) Autokinase activity of full-length CpxA and CpxA_M228V_ proteins as determined using radioactive ATP. It is worth noting that only a small fraction of the CpxA–Brij35 complex (∼0.1%) is phosphorylated at steady state, as estimated by PhosTag gels run under the same conditions used in the radioactive assays (unpublished data).

Despite the overall asymmetry of the CpxA homodimer, the HAMP four-helix bundle is perfectly symmetric as was also observed in the Af1503, Aer2, and VicK structures [15],[28]–[30],[49],[50]. A strict 2-fold axis passing along the length of the coiled-coil relates the two HAMP subunits (Figure 4A), which adopts a packing mode closer to a canonical *knobs-into-holes* model [51] than the unusual *complementary x-da* observed in the wild-type Af1503 structures [28]–[30]. However, a negative slope in the Crick angle deviation plot [29],[52] suggests a departure from an ideal *knobs-into-holes* packing mode due to a weak heptad repeat periodicity at α_1_ (Figure 4B). Overall, the observed CpxA–HAMP conformation resembles that observed in several Af1503 HAMP mutants associated with a kinase-active state [28]–[30].

The similarities in helical packing modes and overall helical bending between CpxA and the Af1503 HAMP–EnvZ DHp fusion chimera (Figure S5) prompted us to investigate whether CpxA follows an analogous two-state mechanism of signal transduction. Based on studies of Af1503, Hulko et al. [30] proposed a model for HAMP-mediated signal transduction, in which the HAMP coiled-coil can adopt two distinct conformations depending on the input signal. In particular, they found that the single point mutation (Ala291–Val) in Af1503 displaced the equilibrium between the two forms [30] and partially reversed the helical bending asymmetry of the wild-type Af1503–EnvZ crystal structure [28]. We therefore decided to introduce the equivalent mutation in CpxA (Ala197–Val; see Figure S2). However, the responses to extracytoplasmic stress due to the overproduction of a folding-defective mutant of MalE in the periplasm (MalE31, [53]), or to the presence of phenethyl alcohol in the inner membrane [54], were similar in bacteria expressing either CpxA_A197V_ or wild-type CpxA (Figure 4C).

To gain further insight into the signaling state(s) of the HAMP domain, we set up a genetic screening to search for mutations that affect the CpxA kinase activity *in vivo*. For this, a plasmid encoding a CpxA variant, which lacks the periplasmic sensor domain (CpxA_ΔP_) and therefore displays constitutive kinase activity, was randomly mutagenized and transformed into a *cpxA*-null strain background. In these cells, the high β-galactosidase activity of the transcriptional fusion (correlated to the kinase activity) provided a means to isolate intragenic suppressors that confer a kinase-deficient/phosphatase-dominant (K^−^/P^+^; i.e., Lac^−^) phenotype, easily monitored on X-gal containing plates. From 48 Lac^−^ colonies examined, the identified mutations were reconstructed into the wild-type *cpxA* gene and their *in vivo* activity was evaluated (Table 2). The strongest phenotype corresponded to the point mutation Met228–Val in the HAMP, which completely abolished the Cpx-system response (Figure 4C). *In vitro* studies revealed that the point mutant CpxA_HDC_M228V_ is less efficient than wild-type CpxA in phosphorylating CpxR (Figure 4D), at least in part as a consequence of a reduced autophosphorylation rate (Figure 4E). Because Met228 occupies a heptad-repeat inner position in the four-helix bundle, its substitution by a smaller valine side-chain would promote a rearrangement of the interhelical packing to avoid the creation of a cavity within the hydrophobic core (Figure 4A). Consistent with a more stable four-helix bundle packing, our SAXS data suggest that the Met228–Val substitution decreases the tendency of CpxA_HDC_ to assemble into tetramers (dimers of dimers), which partially disrupts the overall HAMP structure. Thus, in contrast to wild-type CpxA_HDC_, CpxA_HDC_M228V_ is mainly dimeric in solution, with a relatively constant Rg value at different protein concentrations (Table S1 and Figure S3D).

### A Mechanical Model to Account for Signal Transduction and Autophosphorylation

**Figure 5:**
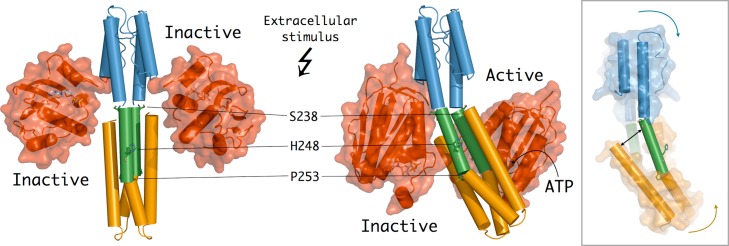
Mechanical model for HK autophosphorylation control. The inactive kinase conformation (left) involves a putative symmetric conformation of CpxA [modeled from the structures of HK853 (PDB ID 2C2A) and wild-type Af1503 HAMP-EnvZ DHp chimera (PDB ID 3ZWR)], in which the two CA domains are sequestered in a nonproductive DHp–CA complex. The active kinase state (right, as observed in the trigonal crystal form) displays a highly asymmetric conformation of the HK homodimer. Propagated by conformational changes in the HAMP domain, the input signal induces a stress on the central DHp helices, promoting segmental helical motions that result in a strong dynamical asymmetry: one of the CA domains is highly mobile and can form a competent active site, whereas the second CA domain is retained in an inactive conformation by extended hydrophobic interactions with the DHp domain. The insert on the right shows these segmental helical movements (without the CA domains for clarity), in which each color represents a distinct rigid-body rotational movement. As a consequence of these movements, a gap broadens between two helices (indicated by a black arrow) and allows the partial exposure of core hydrophobic residues that contribute to sequester the second CA domain in an inactive conformation.

As described above, our biochemical and structural data indicate that HAMP functioning is consistent with a two-state model for signal transduction [49],[55], in which different types of stress (torsional, rotational, or translational) coming from the membrane would break the conformational symmetry of the homodimer by inducing pronounced helical bending of the full CpxA transmitter core and a strong dynamical asymmetry in CA mobility (Figure 5). An important issue here is that, due to the presence of the helix-disrupting points at Ser238 and Pro253 in the long α_2_ helix, the overall bending movement would arise from segmental helical mobility. Thus, the movements of the central part of helix α_2_ (between Ser238 and Pro253, roughly corresponding to the H-box) in each monomer differ from those of the HAMP domain (i.e., α_1_ and N-terminus of α_2_ in both monomers) and the membrane-distal part of the DHp domain (i.e., C-terminus of α_2_ and α_3_ in both monomers), both of which rotate as separate rigid bodies. As a consequence, in the asymmetric (kinase-active) state, the C-terminus of α_3_ in one monomer moves away from the central part of α_2_ in the other monomer, thus partially exposing hydrophobic core residues (Figure 5, insert). This exposure creates a binding interface that retains one CA domain in a fixed inactive conformation (Figure 3D). Instead, on the opposite face of the DHp domain, the same bending movements release the second CA domain, which becomes available to form a transient active site configuration poised for catalysis (Figure 3A). This mechanical model highlights the importance of sequestering the CA domain (through interactions with the DHp domain) as a major mechanism to switch off kinase activity [28], and possibly to bring the catalytic core into phosphatase-active (symmetric) or phosphotransferase-active (asymmetric) states. Taken together, our results point to the importance of conformational and dynamical asymmetry in modulating the autophosphorylation reaction in HKs, in much the same way as ligand-induced asymmetry of homodimeric HK sensor domains was found to play a major role in two-component signal transduction [8],[9].

## Materials and Methods

### Cloning, Expression, and Purification

CpxA_HDC_ construct was cloned into the expression vector pET28a+ (Novagen) and expressed in *E. coli* BLI5 cells. His-tagged CpxA_HDC_ was purified by immobilized metal affinity chromatography followed by gel filtration using standard procedures. For the production of seleno-methionyl (SeMet) CpxA_HDC_, the same plasmid was transformed into the autotrophic *E. coli* B834 (DE3) strain. Differing only in the growth media, SelenoMet Medium (MDL), and L-selenomethionine (50 mg/L) instead of LB, SetMet proteins were purified exactly as the unlabeled ones. Mass spectrometry was used to confirm SeMet incorporation. Full-length CpxA was expressed, solubilized, and purified as described previously [56].

### Crystallization and Data Collection

Purified proteins were concentrated using ultrafiltration VivaSpin devices (30 kDa MWCO, Millipore) to ∼15 mg/ml prior crystallization trials. Crystals were obtained by vapor diffusion at 18°C. All crystal forms grew under similar crystallization conditions [100 mM Tris-HCl (pH 8.5), 1.75 M Ammonium sulphate, and 25% (v/v) glycerol], with the exception of the two monoclinic forms, which were obtained using a solution containing 100 mM Tris-HCl (pH 8.5), 25%(w/v) PEG3350, and 200 mM lithium sulphate as precipitant. In most of the cases, crystals appeared in a few days and reached their maximum size within 2 wk.

Data were collected at ESRF and Soleil synchrotron beamlines (Table 1). Data were indexed and integrated with program *XDS* [57], followed by space group determination with *Pointless*, and scaling with *Aimless* from the *CCP4* software package [58].

### Structure Determination and Refinement

Diffraction data from Se-labeled protein crystals were used to solve the structures of the trigonal (P3_1_21) and hexagonal (P6_1_22) crystal forms. Se atom substructure and phases were estimated using the *AutoSol* wizard of *PHENIX* [59]. After density modification, the resulting electron density maps were readily interpretable for most of the protein chain. Initial models were manually built with *Coot* [60] by fitting into the experimental map template helices and a homology model of the CA domain. The orthorhombic crystal form (C222_1_) was solved by SAD from a Pt derivative dataset at 4.1 Å resolution. Heavy atom positions and phases were estimated using truncated data at 6.5 Å resolution (the anomalous measurability limit) with the *AutoSol* wizard. Despite the poor quality of the experimental map, it was possible to locate the CA domains and build a main-chain trace for the rest of the protein. Given the nonisomorphism between the Pt-derivative and the native dataset at 3.65 Å resolution, an extensive rigid body refinement was necessary to reorient the three dimers present in the native ASU prior to further refinement. Monoclinic crystal forms were determined by molecular replacement with *Phaser* [61] using the previously solved structures as search models. All models were refined with *Buster* [62] including as restrains NCS, TLS, a reference model, and experimental phases when available. Final models were validated with *MolProbity* [63]. Data collection and refinement statistics are shown in Table 1. Figures were generated and rendered with *PyMOL* [64].

### Small Angle X-Ray Scattering

SAXS data were collected at 20°C at beamline BM29 at the ESRF. Data reduction and analysis following the standard procedures were performed using the ATSAS program package [65]. All samples were in 25 mM Tris-HCl buffer, pH 8.5, containing 300 mM NaCl, 5 mM MgCl_2_, 5%(v/v) glycerol, and 0.5 mM TECP.

### Screening for Phosphatase-Dominant (K^−^P^+^) Mutations

Plasmid pLCBA was constructed by subcloning *cpxA* from pIV3cpxA [49] into the low-copy pLCB vector [66]. This plasmid expressed *cpxA* about 1.5-fold in strains grown in LB at 37°C in the absence of arabinose (Betton, unpublished results). The deletion of the periplasmic sensor domain of CpxA was made by the one-step site-directed deletion method [67] using pLCBA as DNA template, and the primers 5′-ATCTAGATTCACCGCTATTACTGCTGATTGTCACCATG-3′ and 5′-TAGATCTAACCCATTGTAGTTTTGGTTGTAGTTGTG-3′. The PCR fragment was first treated with *Dpn*I, and then with *Xba*I before ligation and transformation. The resulting plasmid, pLCBAΔP, encoded a CpxA variant in which the residues 29–163 are substituted by NLDS. Error-prone PCR was performed using the GeneMorph II Random Mutagenesis kit (Agilent Technologies) according to the manufacturer's instructions. Amplification using about 325 ng of *cpxA*Δ*P* gene as DNA template and 20 thermal cycles was performed to aim for 1∼3 mutations/kb. The purified PCR fragment was digested, ligated into pLCB, and transformed into NS54 *E. coli* strain. Approximately 20,000 transformants were platted on LB-agar containing X-gal and 48 white colonies were picked for confirming the linkage of the Lac^−^ phenotype to the plasmid. After DNA sequencing, single suppressor mutations were moved by site-directed mutagenesis on the wild-type *cpxA* gene cloned into pLCBA. To validate the genetic analysis, β-galactosidase activities conferred by the single mutant *cpxA* alleles were assayed *in vivo* in NS54 strain.

### β-Galactosidase Assays

NS54 cells harboring the various pLCB derivatives were grown overnight in LB medium containing chloramphenicol at 37°C, subcultured (1∶100) into 5 ml of the same medium, and then grown to midlog phase at 37°C. The β-galactosidase activity of permeabilized cells was assayed as described previously [53]. A minimum of four independent determinations was averaged to obtain the indicated values.

### Autophosphorylation and Phosphotransfer Assays

Autokinase activity on soluble CpxA_HDC_ and phosphotransfer from full-length CpxA to CpxR_N_ (receiver domain) were assayed by Phos-tag acrylamide gel electrophoresis. Autokinase activity assays on full-length CpxA (as Brij35 complexes) were performed using radioactive ATP as described [56]. For phosphotransfer assays, full-length CpxA (10 µM) in buffer H (25 mM HEPES, pH 7.8, containing 100 mM NaCl, 50 mM KCl, 0.05% Brij35, and 5 mM MgCl_2_) was phosphorylated with 100 µM ATP at 25°C for 20 min. Then, purified CpxR_N_ was added to the reaction to a final concentration of 20 µM. Samples were withdrawn at various time intervals, mixed with SDS sample buffer, and kept on ice until all reactions were completed. Phospho-proteins were separated by Phos-tag acrylamide gel electrophoresis. Polyacrylamide gels were polymerized with 25 µM Phos-tag acrylamide and 50 µM MnCl_2_. Following electrophoresis, gels were stained with Coomassie blue and analyzed by densitometry using the software Quantity One (Biorad). Data were fitted using a nonlinear regression with program Kaleidagraph (Synergy Software).

### Accession Numbers

The crystal structures of CpxA-nucleotide complexes described in this article have been deposited in the Protein Data Bank under accession numbers 4BIU (CpxA_HDC_–ADP, orthorhombic C222_1_ form), 4BIV (CpxA_HDC_–ATP, trigonal P3_1_21 form), 4BIW (CpxA_HDC_M228V_–AMPPNP, hexagonal P6_1_22 form), 4BC0 (CpxA_HDC_–ATP, hexagonal P6_1_22 form), 4BIX (CpxA_HDC_M228V_–ADP, monoclinic C2 form), and 4BIY (CpxA_HDC_M228V_–ADP, monoclinic I2 form).

## Supporting Information

Figure S1 (A) The trigonal crystal form was solved by SAD using Se-labeled protein crystals. Anomalous difference Fourier electron density map (yellow mesh) is contoured at the 5 σ level. Selenomethionine residues are shown as sticks. (B) MR-SAD density modified electron density map (blue mesh) calculated using structure factor anomalous differences and a partial model omitting the HAMP (residues 188–237). Final model is superposed to this unbiased electron density map contoured at the 1.5 σ level.
(TIF)

Figure S2 Structural superposition of the CpxA HAMP domain with those of Af1503 [29] and VicK [15]. The two mutated residues in CpxA, Ala197 (equivalent to Af1503 Ala291) and Met228, are indicated.
(TIF)

Figure S3 (A) Tetrameric form of CpxA (dimer of dimers) as observed in the hexagonal and monoclinic crystal forms. Formation of these tetramers was found to sterically interfere with the positioning of HAMP helix α_1_. (B) Experimental SAXS curves for wild-type CpxA_HDC_ at different protein concentrations in the presence of 5 mM ADP. (C) Same for the point mutant CpxA_HDC_M228V_. (D) Linear dependence of the radius of gyration (Rg) as a function of protein concentration for CpxA_HDC_ (green circles) and CpxA_HDC_M228V_ (blue circles).
(TIF)

Figure S4 Cartoon representation of the ATP-binding CA domain. Highlighted in colors are the highly conserved sequence motifs (G1, G2, N, and F boxes) in the GHKL superfamily.
(TIF)

Figure S5 A similar helical core bending was observed in the crystal structures of CpxA_HDC_ (left) and wild-type Af1503 HAMP–EnvZ DHp chimera (right).
(TIF)

Figure S6 Structural superposition reveals that the conformation of the catalytic core of wild-type CpxA_HDC_ in complex with ATP (yellow) crystallized in the trigonal space group P3_1_21 is very similar to that observed in the hexagonal (P6_1_22) crystal form for either the wild-type CpxA_HDC_–ATP (pink) or the point mutant CpxA_HDC_M228V_–AMPPNP (green) complexes. The catalytic core (residues 238–455) of CpxA_HDC_–ATP (trigonal form) and CpxA_HDC_M228V_–AMPPNP (hexagonal form) crystal structures can be superposed with an RMSD of 1.36 Å. More significant changes are observed, however, for the N-terminal helix of the HAMP domain due to different crystal packing environments.
(TIF)

Figure S7 Protein–nucleotide hydrogen-bonding interactions as observed in the crystal structure of CpxA_HDC_ in complex with AMPPNP (hexagonal crystal form).
(TIF)

Figure S8 The structural superposition of CpxA DHp domain (in green) and CheA P1 domain (PDB ID 1TQG, in yellow) reveals a spatially equivalent glutamic acid that makes hydrogen-bonding interactions with Nδ of the catalytic histidine and serves to enhance nucleophilic catalysis. However, the two proteins display a different topology: whereas the two residues in CpxA are part of the conserved H-box in helix α2, they belong to different helices in CheA.
(TIF)

Figure S9 Cartoon and schematic representations of the DHp four-helix bundle assembly and CA domain positioning in (A) *trans*-autophosphorylating CpxA_HDC_ (hexagonal crystal form) and (B) *cis*-autophosphorylating VicK (4I5S) crystal structures.
(TIF)

Figure S10 Half occupation of the ATP binding sites. Cartoon representation of the single occupied ADP-binding site in the CpxA_HDC_M228V_ homodimer crystallized in the monoclinic C2 crystal form (the second monomer in this crystal structure has no bound nucleotide). The green mesh corresponds to a 2 Å resolution σ_A_ weighted difference electron density map (mFo-DFc) contoured at the 3 σ level. The ADP molecule and the Mg^2+^ ion (shown as ball-and-sticks) were omitted from the model before map calculation. Also shown are residues Phe403 and Leu419 engaged in the CA–DHp interface. It can be argued that the presence of ADP (instead of ATP or AMPPNP) in the crystals might account for the observed half-occupancy. Against this hypothesis, however, the structure of CpxA_HDC_ in complex with AMPPNP (determined at 2.7 Å resolution in the same monoclinic C2 crystal form; unpublished data) showed the same half-occupancy pattern.
(TIF)

Table S1 CpxA_HDC_ and CpxA_HDC_M228V_ SAXS-derived parameters.
(DOCX)

## Abbreviations

AMP-PNP: Adenosine 5′-(β,γ-imido)triphosphate
CA: catalytic and ATP binding domain; DHp, dimerization and histidine phosphorylation
HAMP: Histidine kinase, Adenyl cyclase, Methyl-accepting proteins, Phosphatase
HK: histidine kinase
RR: response regulator
TCS: two-component system
