# Supplementary figures and images for: Segmental Helical Motions and Dynamical Asymmetry Modulate Histidine Kinase Autophosphorylation
Source: PLoS Biol. 2014 Jan 28;12(1):e1001776. doi: 10.1371/journal.pbio.1001776 (PMC3904827; doi:10.1371/journal.pbio.1001776)

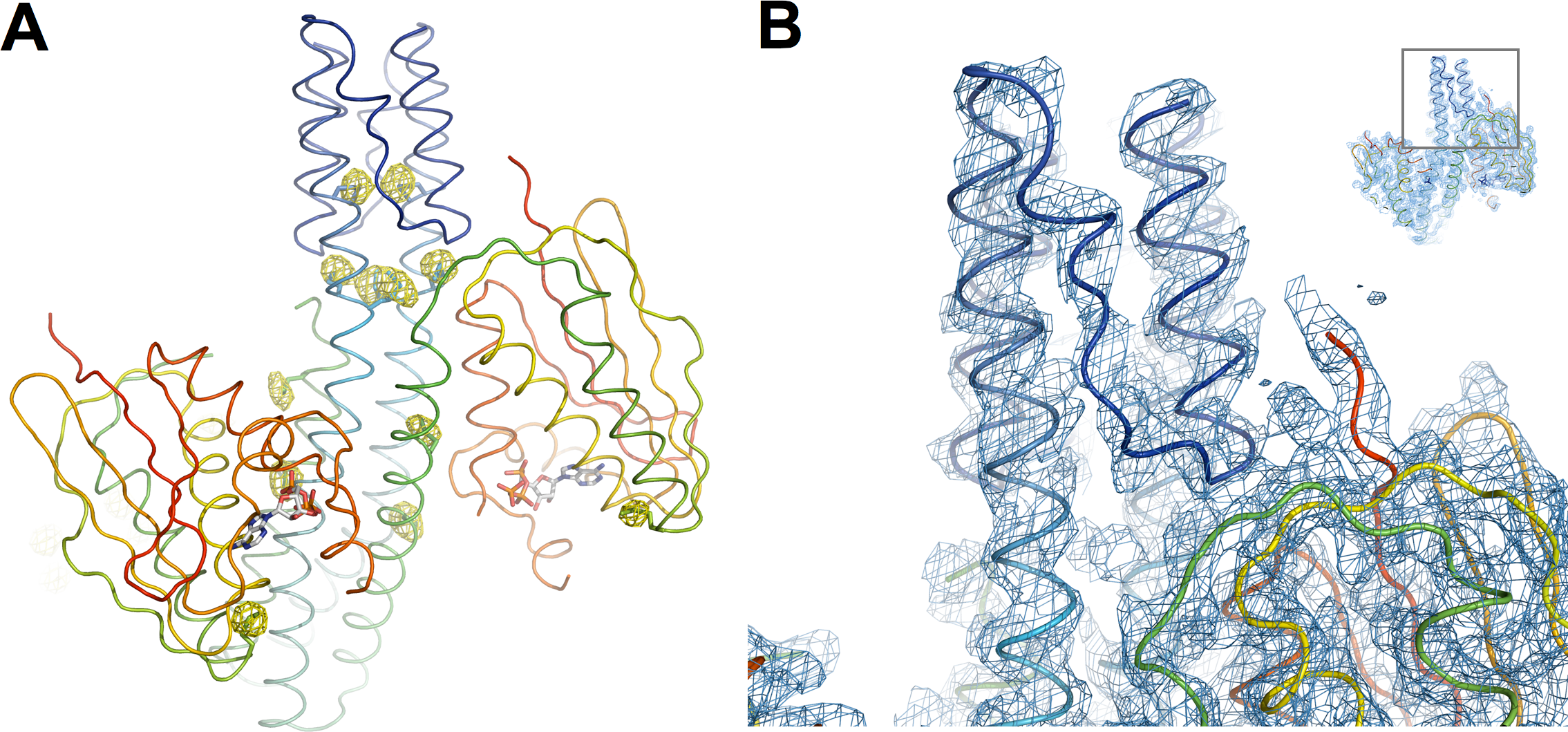

Supplement: Figure S1 — (A) The trigonal crystal form was solved by SAD using Se-labeled protein crystals. Anomalous difference Fourier electron density map (yellow mesh) is contoured at the 5 σ level. Selenomethionine residues are shown as sticks. (B) MR-SAD density modified electron density map (blue mesh) calculated using structure factor anomalous differences and a partial model omitting the HAMP (residues 188–237). Final model is superposed to this unbiased electron density map contoured at the 1.5 σ level. (TIF) [file pbio.1001776.s001.tif]

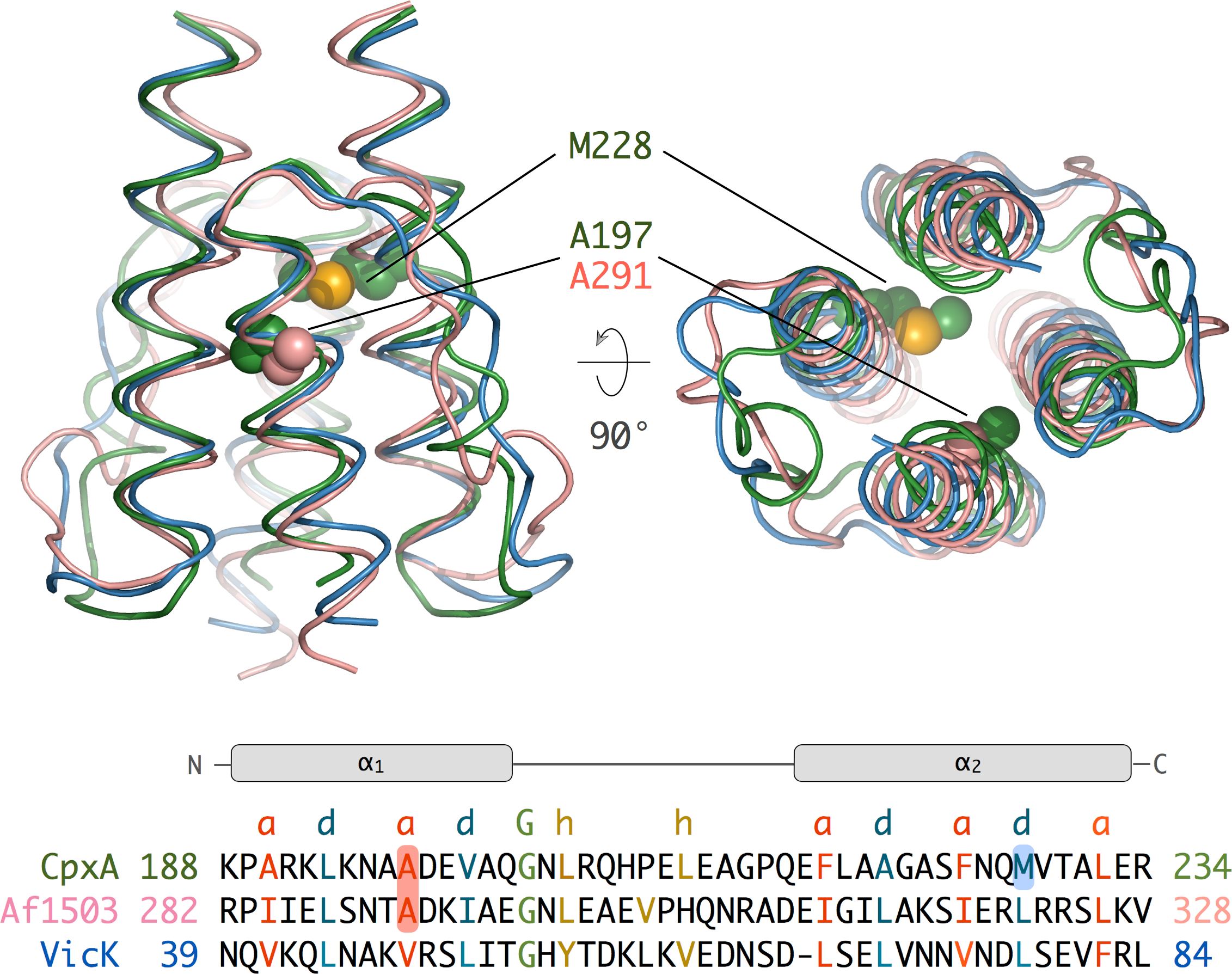

Supplement: Figure S2 — Structural superposition of the CpxA HAMP domain with those of Af1503 [29] and VicK [15]. The two mutated residues in CpxA, Ala197 (equivalent to Af1503 Ala291) and Met228, are indicated. (TIF) [file pbio.1001776.s002.tif]

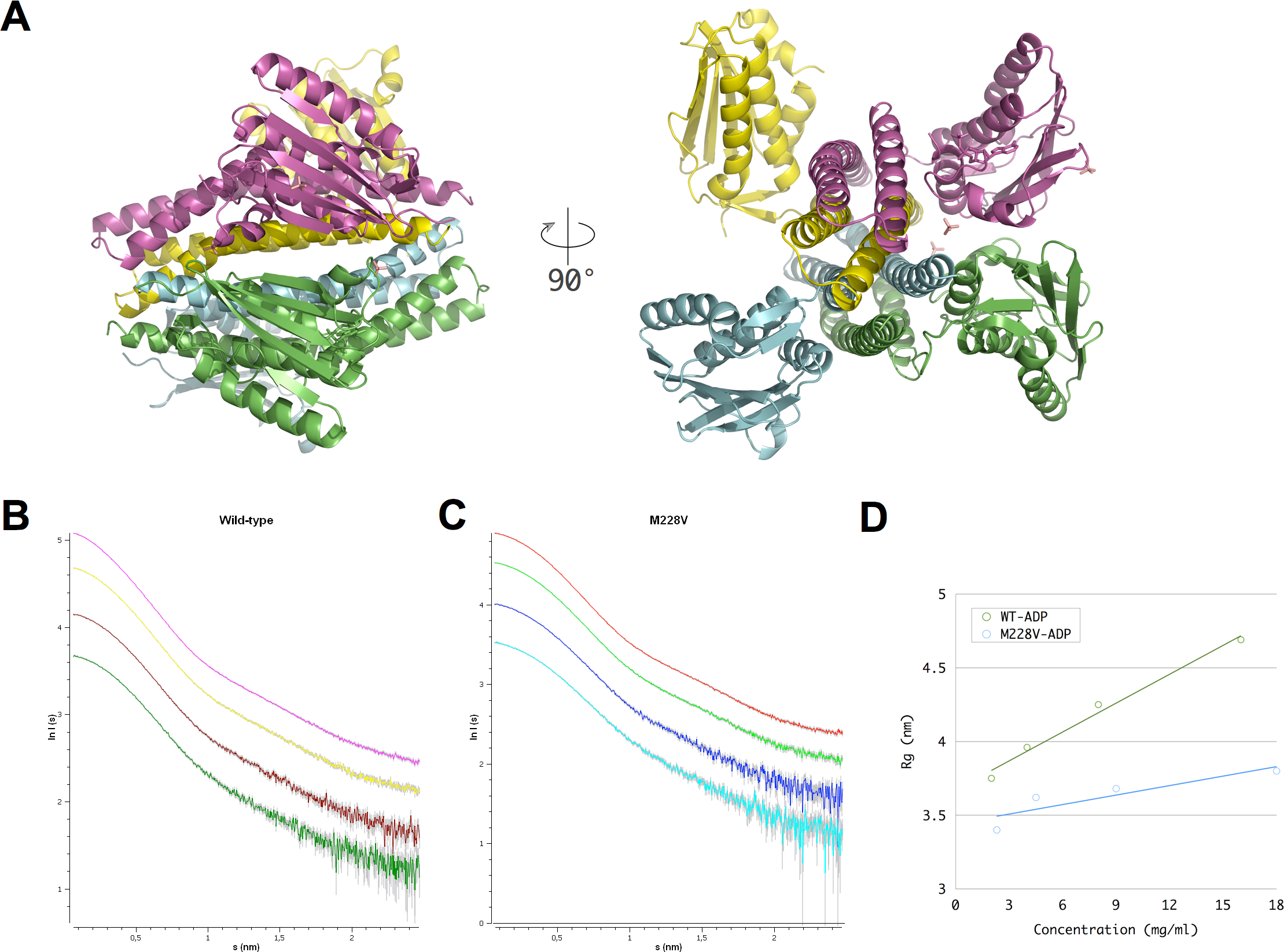

Supplement: Figure S3 — (A) Tetrameric form of CpxA (dimer of dimers) as observed in the hexagonal and monoclinic crystal forms. Formation of these tetramers was found to sterically interfere with the positioning of HAMP helix α1. (B) Experimental SAXS curves for wild-type CpxAHDC at different protein concentrations in the presence of 5 mM ADP. (C) Same for the point mutant CpxAHDC_M228V. (D) Linear dependence of the radius of gyration (Rg) as a function of protein concentration for CpxAHDC (green circles) and CpxAHDC_M228V (blue circles). (TIF) [file pbio.1001776.s003.tif]

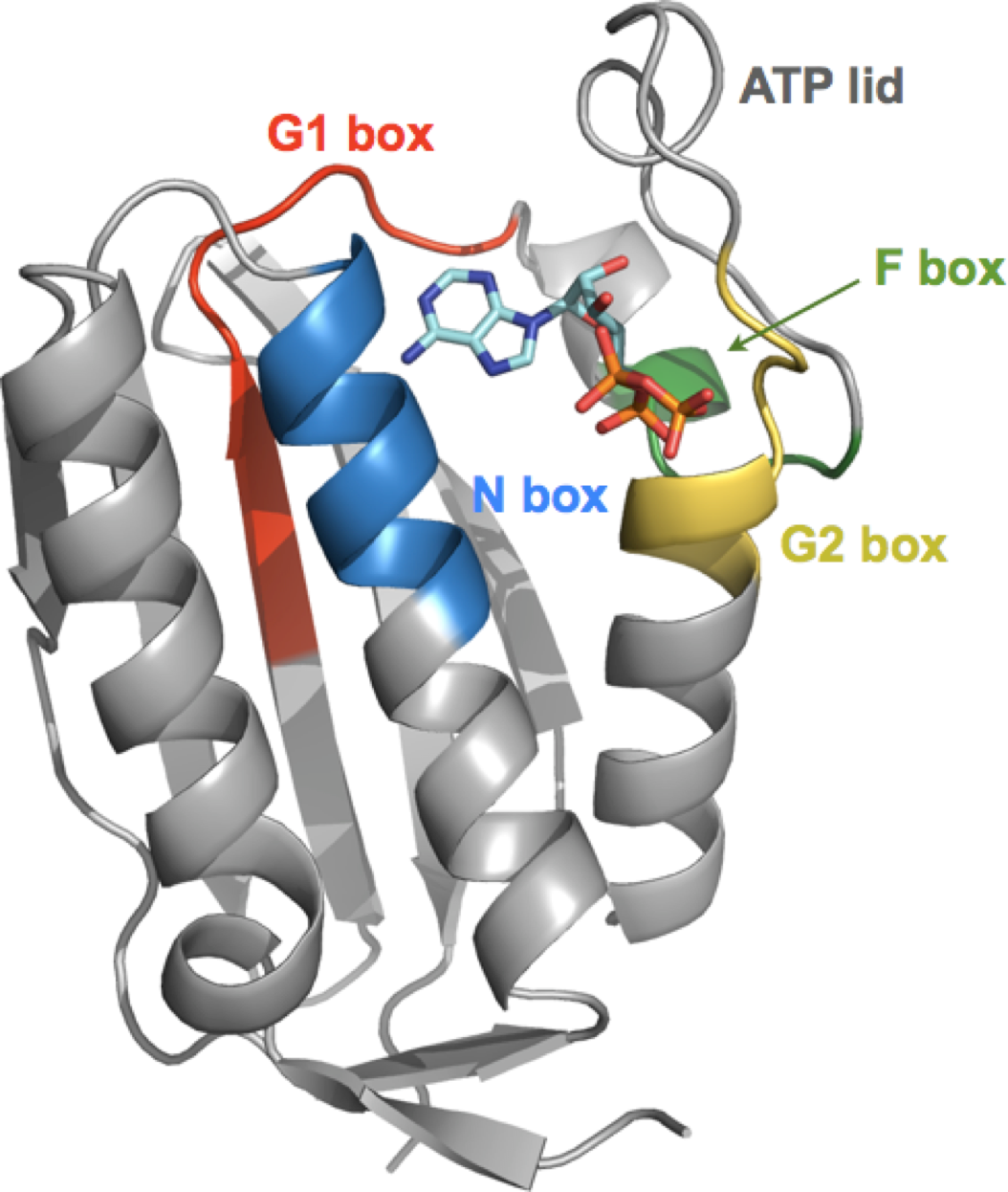

Supplement: Figure S4 — Cartoon representation of the ATP-binding CA domain. Highlighted in colors are the highly conserved sequence motifs (G1, G2, N, and F boxes) in the GHKL superfamily. (TIF) [file pbio.1001776.s004.tif]

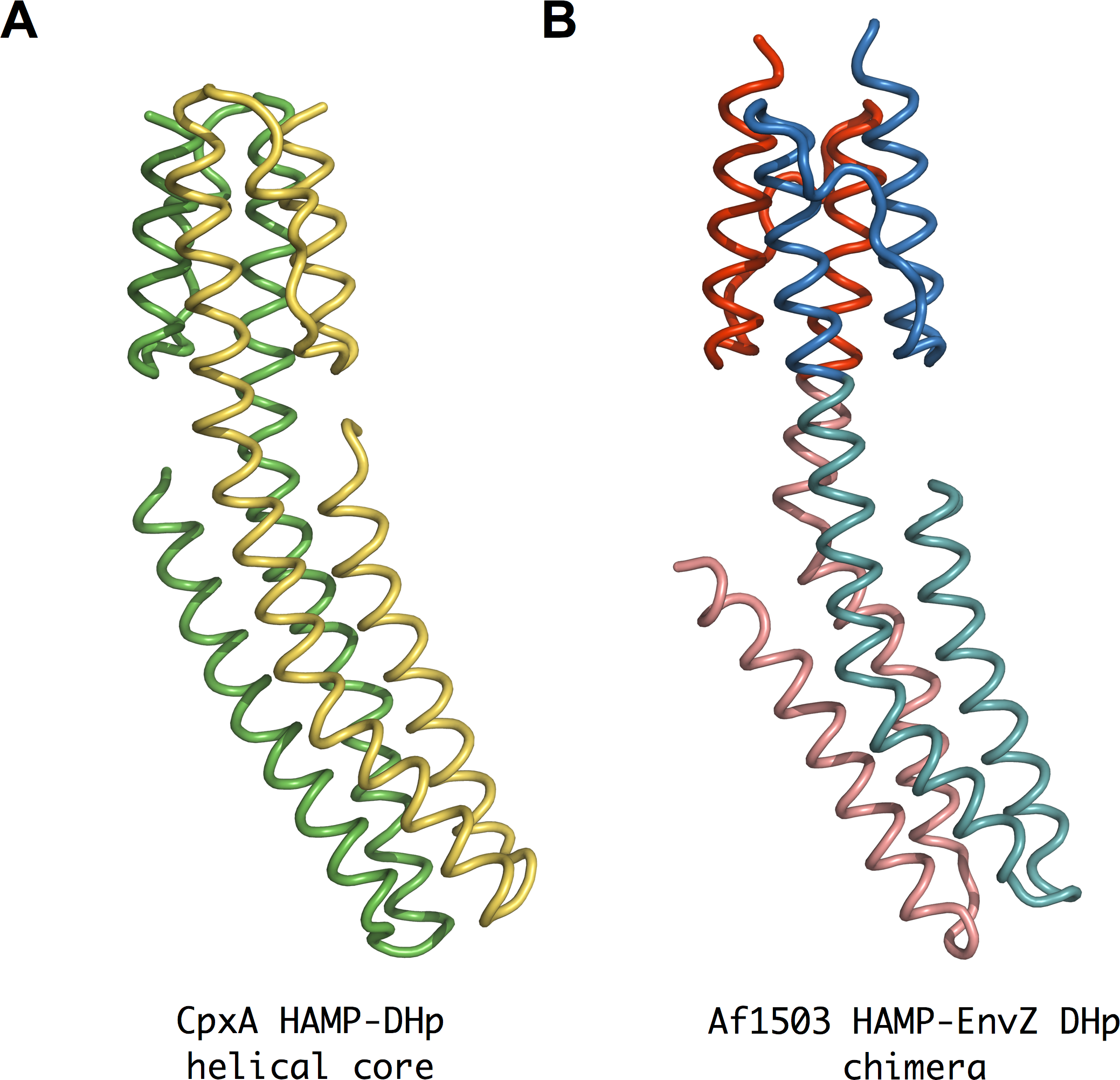

Supplement: Figure S5 — A similar helical core bending was observed in the crystal structures of CpxAHDC (left) and wild-type Af1503 HAMP–EnvZ DHp chimera (right). (TIF) [file pbio.1001776.s005.tif]

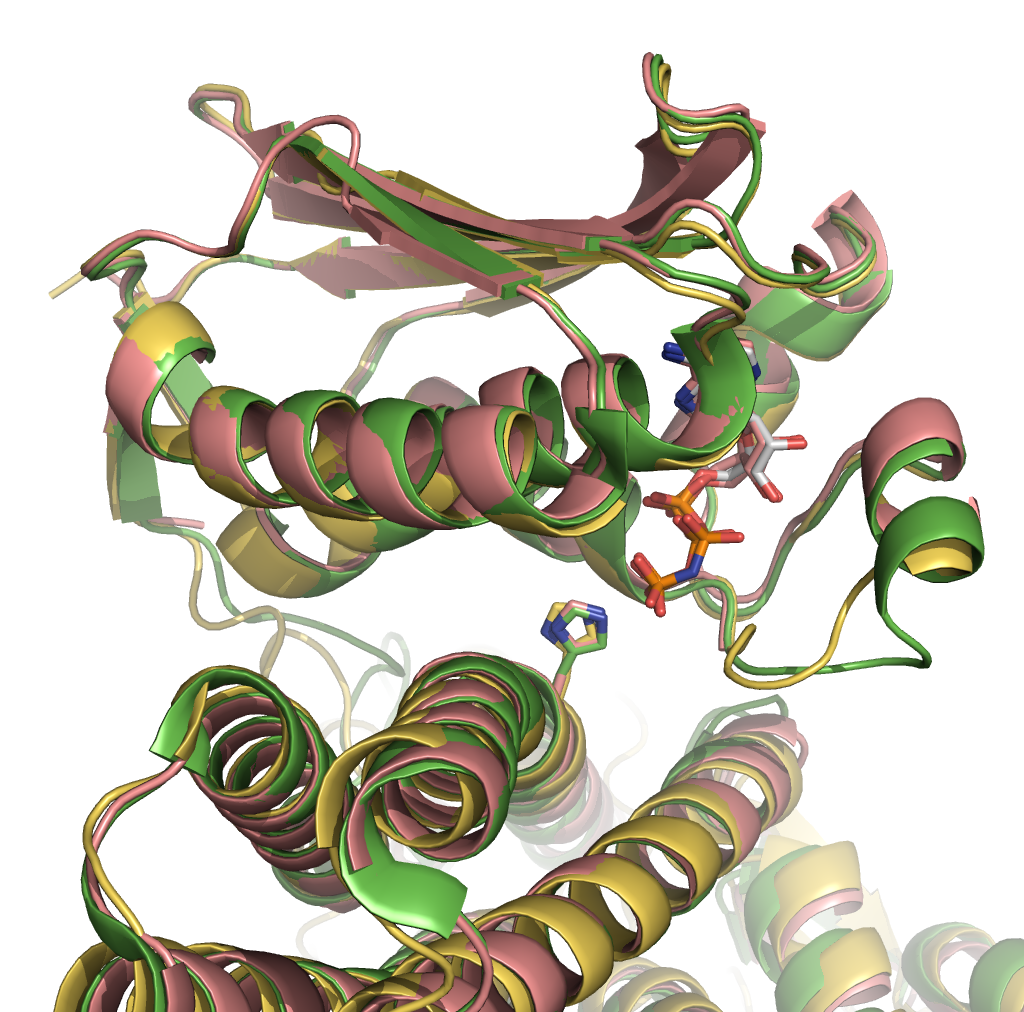

Supplement: Figure S6 — Structural superposition reveals that the conformation of the catalytic core of wild-type CpxAHDC in complex with ATP (yellow) crystallized in the trigonal space group P3121 is very similar to that observed in the hexagonal (P6122) crystal form for either the wild-type CpxAHDC–ATP (pink) or the point mutant CpxAHDC_M228V–AMPPNP (green) complexes. The catalytic core (residues 238–455) of CpxAHDC–ATP (trigonal form) and CpxAHDC_M228V–AMPPNP (hexagonal form) crystal structures can be superposed with an RMSD of 1.36 Å. More significant changes are observed, however, for the N-terminal helix of the HAMP domain due to different crystal packing environments. (TIF) [file pbio.1001776.s006.tif]

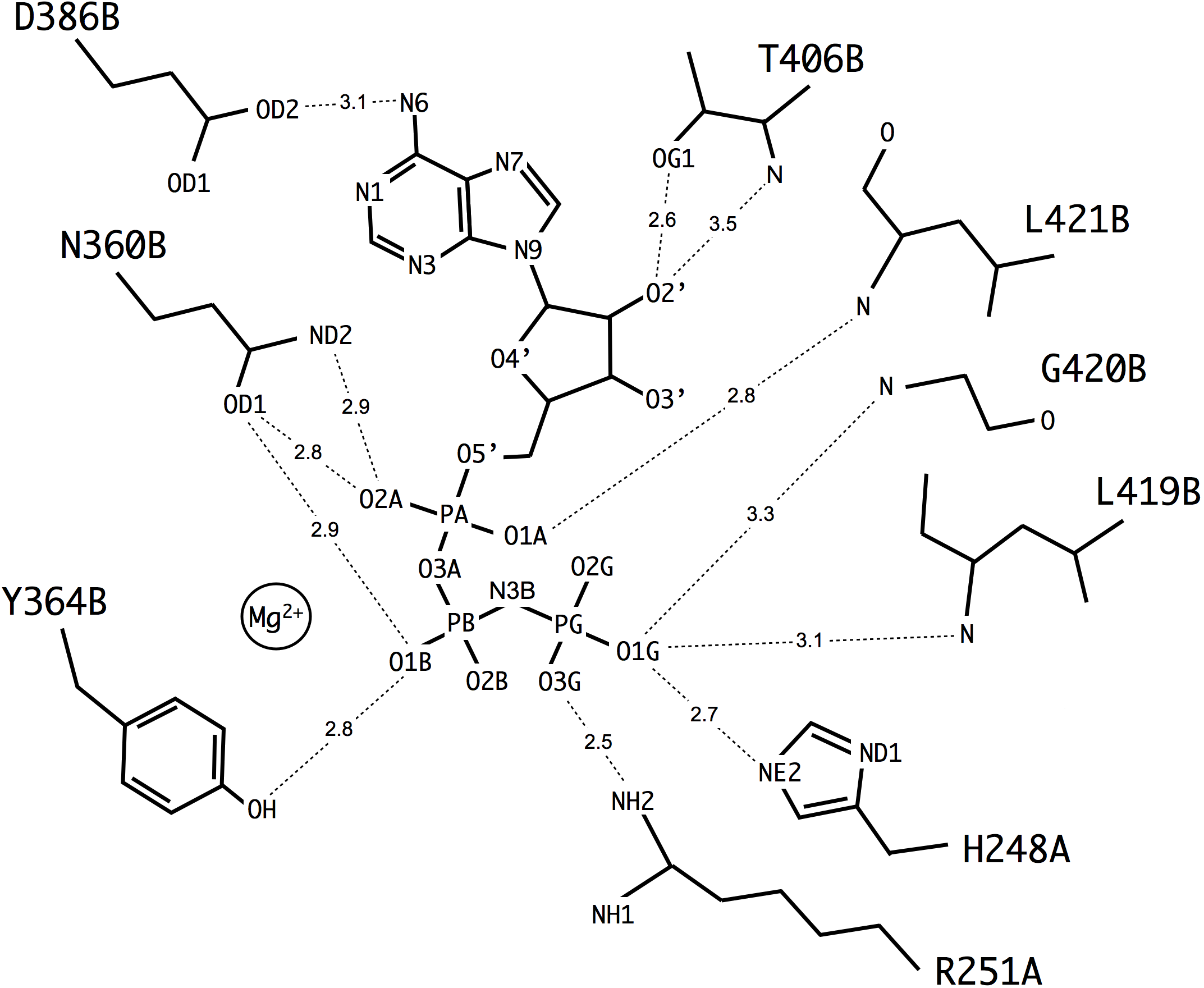

Supplement: Figure S7 — Protein–nucleotide hydrogen-bonding interactions as observed in the crystal structure of CpxAHDC in complex with AMPPNP (hexagonal crystal form). (TIF) [file pbio.1001776.s007.tif]

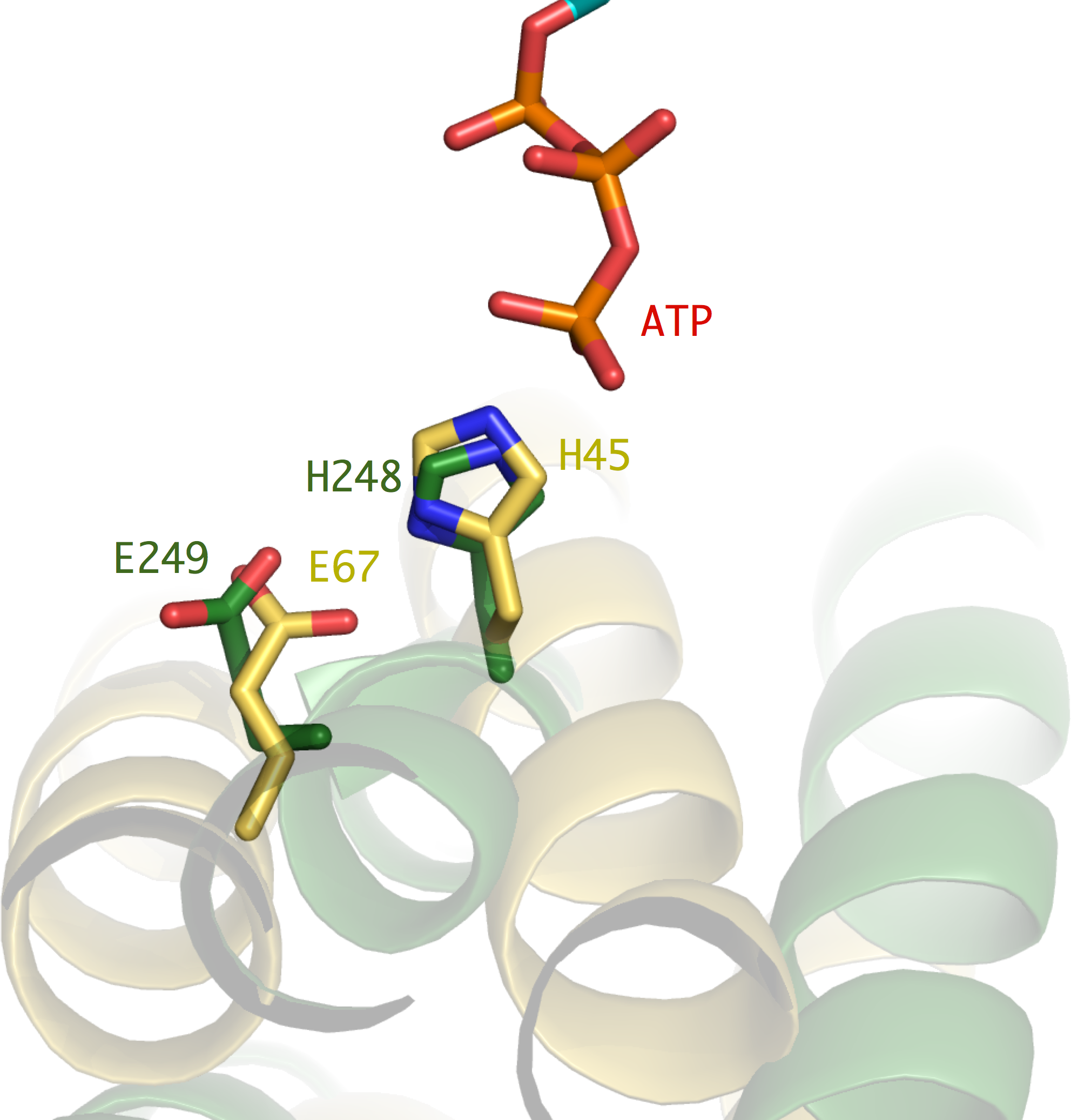

Supplement: Figure S8 — The structural superposition of CpxA DHp domain (in green) and CheA P1 domain (PDB ID 1TQG, in yellow) reveals a spatially equivalent glutamic acid that makes hydrogen-bonding interactions with Nδ of the catalytic histidine and serves to enhance nucleophilic catalysis. However, the two proteins display a different topology: whereas the two residues in CpxA are part of the conserved H-box in helix α2, they belong to different helices in CheA. (TIF) [file pbio.1001776.s008.tif]

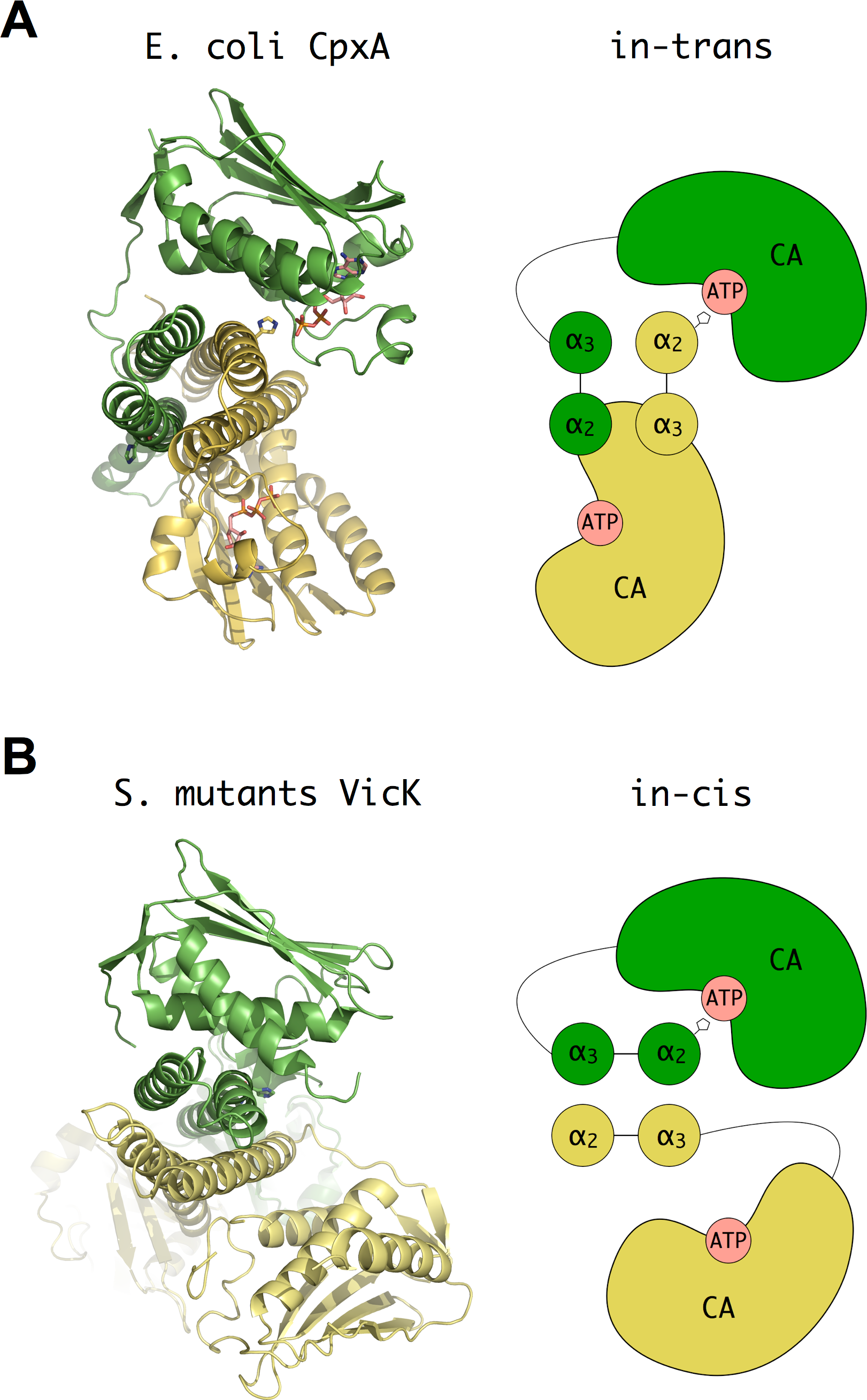

Supplement: Figure S9 — Cartoon and schematic representations of the DHp four-helix bundle assembly and CA domain positioning in (A) trans-autophosphorylating CpxAHDC (hexagonal crystal form) and (B) cis-autophosphorylating VicK (4I5S) crystal structures. (TIF) [file pbio.1001776.s009.tif]

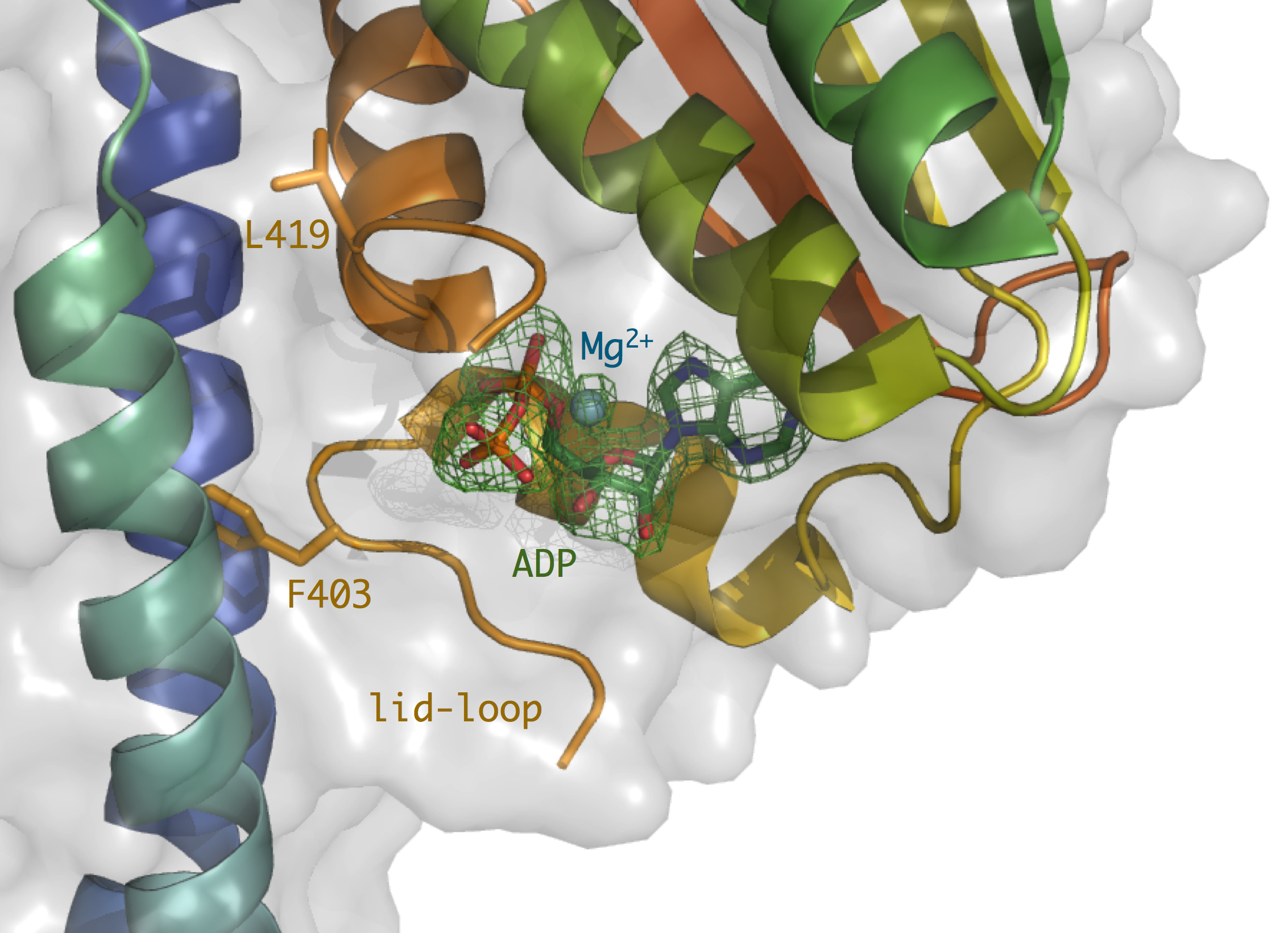

Supplement: Figure S10 — Half occupation of the ATP binding sites. Cartoon representation of the single occupied ADP-binding site in the CpxAHDC_M228V homodimer crystallized in the monoclinic C2 crystal form (the second monomer in this crystal structure has no bound nucleotide). The green mesh corresponds to a 2 Å resolution σA weighted difference electron density map (mFo-DFc) contoured at the 3 σ level. The ADP molecule and the Mg2+ ion (shown as ball-and-sticks) were omitted from the model before map calculation. Also shown are residues Phe403 and Leu419 engaged in the CA–DHp interface. It can be argued that the presence of ADP (instead of ATP or AMPPNP) in the crystals might account for the observed half-occupancy. Against this hypothesis, however, the structure of CpxAHDC in complex with AMPPNP (determined at 2.7 Å resolution in the same monoclinic C2 crystal form; unpublished data) showed the same half-occupancy pattern. (TIF) [file pbio.1001776.s010.tif]
