# Supplementary material for: Segmental Helical Motions and Dynamical Asymmetry Modulate Histidine Kinase Autophosphorylation
Source: PLoS Biol. 2014 Jan 28;12(1):e1001776. doi: 10.1371/journal.pbio.1001776 (PMC3904827; doi:10.1371/journal.pbio.1001776)
Supplement: Table S1 — CpxAHDC and CpxAHDC_M228V SAXS-derived parameters. (DOCX) [file pbio.1001776.s011.docx]

| Sample description | Conc.  (mg/ml) | R_g_  (nm) | I(0) | D_max_  (nm) | MM  (kDa) | Volume  nm^3^ |
| --- | --- | --- | --- | --- | --- | --- |
| CpxA_HDC_ | 16.0 | 4.69 | 124.3 | 16.4 | 119.0 | 251.0 |
|  | 8.0 | 4.25 | 109.5 | 14.9 | 105.0 | 259.0 |
|  | 4.0 | 3.96 | 96.6 | 13.9 | 92.0 | 179.0 |
|  | 2.0 | 3.75 | 95.3 | 13.1 | 91.0 | 210.0 |
| CpxA_HDC_M228V_ | 18.0 | 3.80 | 80.1 | 13.3 | 77.0 | 166.0 |
|  | 9.0 | 3.68 | 75.4 | 12.9 | 72.0 | 194.0 |
|  | 4.5 | 3.62 | 68.7 | 12.7 | 66.0 | 176.0 |
|  | 2.3 | 3.40 | 66.3 | 11.9 | 63.0 | 160.0 |
